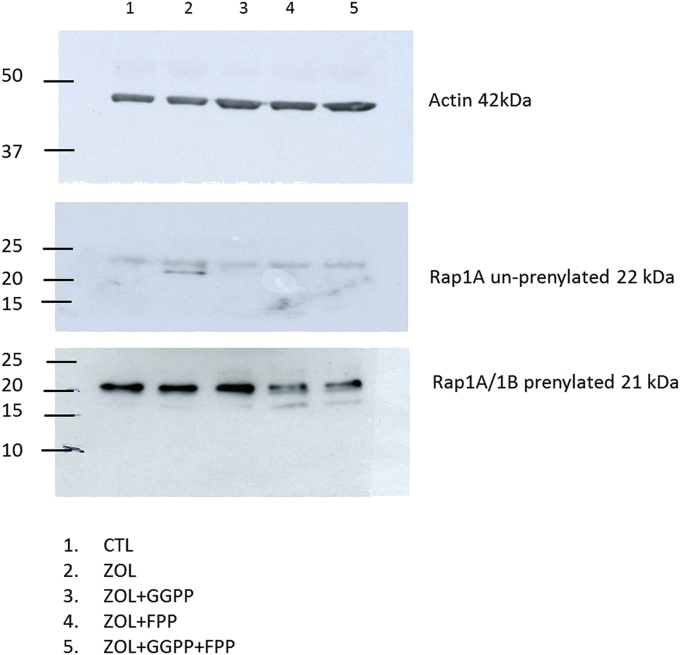# Zoledronic acid inhibits the growth of leukemic MLL-AF9 transformed hematopoietic cells

**DOI:** 10.1016/j.heliyon.2020.e04020

**Published:** 2020-06-05

**Authors:** Emanuela Chiarella, Bruna Codispoti, Annamaria Aloisio, Emanuela G. Cosentino, Stefania Scicchitano, Ylenia Montalcini, Daniela Lico, Giovanni Morrone, Maria Mesuraca, Heather M. Bond

**Affiliations:** a Laboratory of Molecular Haematopoiesis and Stem Cell Biology, Dept. of Experimental and Clinical Medicine, University Magna Græcia, 88100 Catanzaro, Italy; b Tecnologica Research Institute-Marrelli Health, 88900 Crotone, Italy; c Exiris S.r.l., 00128 Roma, Italy; d Department of Obstetrics & Ginecology, University Magna Græcia, 88100 Catanzaro, Italy

**Keywords:** Biological sciences, Cell biology, Biochemistry, Molecular biology, Cancer research, Acute myeloid leukemia, MLL-AF9 gene rearrangement, Bisphosphonate, Zoledronic acid

## Abstract

A leukemic *in vitro* model produced by transducing Cord Blood derived-hematopoietic CD34^+^ cells with the MLL-AF9 translocation resulting in the oncogenic fusion protein, is used to assess for sensitivity to Zoledronic acid. These cells are practically immortalized and are of myeloid origin. Proliferation, clonogenic and stromal co-culture assays showed that the MLL-AF9 cells were considerably more sensitive to Zoledronic acid than normal hematopoietic CD34^+^ cells or MS-5 stromal cells. The MLL-AF9 cells were notably more inhibited by Zoledronic acid when cultured as colonies in 3 dimensions, requiring cell-cell contacts compared to suspension expansion cultures. This is coherent with the mechanism of action of Zoledronic acid inhibiting farnesyl diphosphate synthase which results in a block in prenylation of GTPases such that their role in the membrane is compromised for cell-cell contacts. Zoledronic acid can be proposed to target the MLL-AF9 leukemic stem cells before they emerge from the hematopoietic niche, which being in proximity to bone osteoclasts where Zoledronic acid is sequestered can be predicted to result in sufficient levels to result in an anti-leukemic action.

## 1 Introduction

Zoledronic acid (ZOL) is a third-generation bisphosphonate, commonly used for the treatment and prevention of osteoporosis [1] as well as for metastatic bone disease [2]. Bisphosphonates accumulate in bones by binding to hydroxyapatite crystals, and act on osteoclasts inhibiting resorption. The fact that ZOL is highly absorbed by the bone matrix, but not by other tissues, makes it an ideal candidate for the treatment of bone diseases. Bisphosphonates act in the mevalonate pathway by inhibiting farnesyl diphosphate synthase (FDPS) [3], which is required for the biosynthesis of cholesterol and as well as other isoprenoid lipids such as farnesyl pyrophosphate (FPP) and geranylgeranyl pyrophosphate (GGPP). These molecules are responsible for the prenylation of small GTPases (Ras, Rho, Rac) that, in turn, regulate osteoclast function and can be activated in leukemias and hematological malignancies [4].

In this manuscript we have addressed the question of whether ZOL could be effective in leukemias with the MLL-AF9 rearrangement where in particular the Rac-GTPase signaling is found to be required for leukemic cell growth. The rational is that ZOL can block the key enzyme required for farnesylation needed for prenylation of the GTPases thus preventing association with the plasma membrane for Rac signaling.

ZOL is currently approved and used for the treatment of bone metastasis in renal, breast and prostate advanced cancer patients, but its role in other tumours and leukemias remains debatable. Concerning leukemias, ZOL has been used for the control of calcium levels in childhood leukemic patients who can sometimes have hypercalcemia. Indeed hypercalcemia can be an early sign of childhood ALL and has in specific cases been successfully treated with ZOL [5, 6], reducing calcium levels in the serum, as ZOL is adsorbed to the surface of bone hydroxyapatite and inhibiting calcium release by interfering with osteoclast-mediated bone resorption. The combination of therapy with ATRA and itraconazole [7] in an acute promyelocytic leukemia APL patient led to hypercalcemia and was successfully treated with ZOL reducing calcium levels. Treatment with ZOL for the control of hypercalcemia has not always been successful [8] in a case of juvenile myelomonocytic leukemia (JMML) with PTPN11 mutation where the RAS pathway was dysregulated, this may however be due to the progression of the leukemia at the late time of treatment.

ZOL has also been used to prophylactically treat AML patients to prevent the potential development of osteopenia and osteoporosis arising after allogenic stem cell transplantations. This therapeutic strategy was found to be safe and promising for limiting bone loss without any signs of osteonecrosis of the jaw [9].

The bone marrow microenvironment is understood to be modulated when malignant cells arise, this can in pre B-ALL patients result in significant bone deterioration. Based on a mouse model it has been shown that the addition of ZOL to therapy could rescue leukemia-induced bone loss, reduce disease burden and prolong survival in leukemia-bearing mice [10].

When ZOL was primarily used to treat leukemic patients who develop osteoporosis or hypercalcemia, its action is aimed at its role in bone homeostasis rather than specifically at proliferating leukemic blasts. The use of leukemic cell lines has shown an inhibitory effect of ZOL in proliferation, colony formation and apoptosis assays [11, 12, 13, 14]. *In vitro* studies with patient derived leukemic blasts have demonstrated that ZOL can be have a direct effect. Freshly isolated blasts from leukemic AML patients were used to show that ZOL has the potential to block proliferation and induce apoptosis [11] and that this cytotoxic effect was additive with the chemotherapeutic drug cytarabine. Selective sensitive to ZOL was not confined to cases with RAS activation.

Juvenile myelomonocytic leukemic cells are often characterised by having activated GM-CSF signaling *via* the RAS pathway, this was targeted with ZOL impairing *in vitro* colony formation. Leukemic cell cultures displayed decreased proliferation and monocyte/macrophage differentiation whereas normal bone marrow cultures were relatively unaffected [15]. *In vitro* assays using cell lines with activated RAS related proteins owing to Bcr/abl Ph^+^ have shown that ZOL especially with imatinib mesylate can result in increased survival in mice [16] and in patient derived Bcr/abl leukemic cells (ALL and CML) inoculated into mice, a higher sensitivity due to the combination of ZOL and imatinib mesylate [17]. CML patients can be resistant to imatinib owing to overexpression of Bcr-abl and upregulation of P-glycoprotein in these cases ZOL was still effective in inhibiting proliferation and clonogenicity in patient derived cells [18].

Given the close proximity of the hematopoietic niche with bone osteoblasts, studies have been performed to evaluate the effect of ZOL in mice models, where ZOL was found in addition to increasing bone volume and blood vessel numbers, able to induce HSCs expansion indirectly through the osteoblastic niche [19]. Breast tumor mouse models were used to show that ZOL increased the endosteal and vascular niche as well as inducing a transient increase in hematopoietic cells and inhibition of breast tumor outgrowth *in vivo* [20].

An indirect anti-tumorigenicity role for ZOL could be demonstrated through its ability to stimulate the immune system. ZOL inhibits the farnesyl pyrophosphate synthase in the mevalonate pathway of cholesterol synthesis, leading to an upstream accumulation of isopentenyl pyrophosphate (IPP). This metabolite results in Vδ2 T-cell activation and expansion in the presence of IL-2 [21]. Additionally when the combination of ZOL and immunomodulatory drugs, lenalidomide or pomalidomide were used and there was an expansion of Th1-like Vγ9Vδ2T cells resulting in cytotoxicity against Multiple Myeloma [22].

The present study evaluates the effect of ZOL on acute myeloid leukemia model with the MLL-AF9 (MA9) rearrangement. The mixed lineage leukemia (MLL) gene translocations are associated with poor prognosis. The MLL gene encodes for a methyltransferase protein [23, 24] and when fused with partner proteins, such as AF9, the catalytic domain is lost and the aberrant fusion protein gains the ability to methylate H3K79, which results in abnormal gene expression of genes such as HOXA9 and MEIS1. Immunocompromised mice transplanted with cord blood (CB) cells transformed with the MA9 fusion gene, develop myeloid or lymphoid leukemias [25, 26, 27]. HSCs from foetal origin, transformed with MA9 fusion gene, develop both AML and ALL; instead bone marrow derived transfected HSCs give rise, with inferior efficacy, essentially to AML [28]. These MA9 cells have been found to be sensitive to cholesterol metabolism and the use of statins blocked their growth sparing normal HSCs [29, 30]. Additionally the use of Rac1/2 GTPase inhibitors can specifically inhibit MA9 leukemias [31, 32]. The Rac-GTPases are required in HSCs for their functional activity involving migration, adhesion, survival and retention in the bone marrow niche and are often deregulated in leukemias [4]. The farnesylation/prenylation derived from the isoprenoid intermediates of the mevalonate pathway for these GTPases is required for functional activity in leukemic cells. Here we investigate the effects of ZOL, an inhibitor of FDPS, on CB-HSCs transformed by the MA9 lentivirus (CB-MA9 cells) compared to normal CB-HSCs (CD34^+^ cells) and MS-5 stromal cells and have found a high sensitivity for proliferation, clonogenicity and cobblestone area formation.

## 2 Materials and methods

### 2.1 Cell culture and reagents

CB-CD34^+^ cells were purchased from Lonza and cultured at 0.5–1.0 × 10^5^ cells/ml in xeno-free StemMACS™ HSC Expansion Media (Miltenyi Biotec) in the presence of Thrombopoietin (TPO), FLT3-Ligand (FLT3-L) and Stem Cell Factor (SCF) (100 ng/ml) [33].

CB-CD34^+^ MA9 cells were produced by lentiviral transduction [34, 35]. Briefly, CD34^+^ cells were cultured in StemMACS™ HSC Expansion Media (Miltenyi Biotec) in presence of TPO, FLT3-L and SCF (100 ng/ml). After 24h, 1 × 10^5^ cells were infected using the lentiviral vectors UMG-LV6-MLL/AF9 or control empty vector UMG-LV6 at an MOI of 2 prepared using packaging plasmids (pCMV-ΔR8–91, pCMV-VSVG) and quantified using the K562 cell line [34]. The transduction was enhanced by two rounds of spin-inoculation at 32 °C, 1900 rpm for 50 min, in the presence of 8 μg/ml of polybrene (Sigma). CB-MA9 cells were then washed with PBS and cultured in MyeloCult™ H5100 (STEMCELL Technologies) supplemented with 1 μM of Hydrocortisone HC, FLT3-L, SCF and IL3 (10 ng/ml). Transduction efficiency was evaluated by Q-PCR analysis using primers for the breakpoint of MA9. The UMG-LV6-MA9 vector has the same MA9 translocation as the FEIGW-MA9 vector [28] only having instead of the EF1α promoter, a bidirectional cassette for MA9 controlled by the UBC promoter and EGFP by the minimal regulatory element (170 bp) of the WASP promoter for high EGFP expression. Experiments were performed with 3 different lots of CB-CD34^+^ cells each transduced with MA9 or control vector.

The mouse stromal cell line MS-5 was cultured in Minimum Essential Medium Eagle (Alpha MEM) supplemented with 10% fetal bovine serum, 50U of penicillin and 50 μg/ml of streptomycin. Cells were maintained at 37 °C in a 5% CO_2_, humidified incubator.

The bisphosphonate Zoledronic acid (ZOL) Mylan (4mg/5ml stock) was diluted with medium for assays.

### 2.2 Cell viability assays

CB-MA9 cells (2 × 10^3^) were plated in 100 μl of complete MyeloCult with the RealTime-Glo MT Cell Viability Substrate (Promega) and treated with increasing concentrations of ZOL. Luminescence was assayed by the GloMax EXPLORER (Promega). Cells not treated with ZOL were used as controls. Cell viability was evaluated in quadruplicate.

### 2.3 Colony-forming assay

CB-MA9 cells were plated in 4 well dishes in StemMACS HSC-CFU complete with Epo (Miltenyi Biotec) with 1% methylcellulose and 10% FBS. After gently vortexing cells were plated at 500 μL methylcellulose/well using a 18-gauge blunt needle. After 14 days, the colonies formed were scored and representative images were captured with an inverted microscope EVOs (Life Technologies) at 10x magnification. Colony sizes were estimated by diameter measurements using ImageJ analysis. Assays were performed in duplicate.

### 2.4 Assessment of cobblestone areas (CAs)

CB-CD34^+^ or CB-MA9 cells were cultured on a monolayer of MS-5 stromal cells (previously treated with mitomycin C, 10 μg/ml) in 96 well plates in MyeloCult H5100 and treated with increasing ZOL concentrations. For assessment of early CAs after 6 days of culture, wells were scored microscopically for the presence of small embedded “cobblestone like” cells. Images were captured with a DFC3000 G camera mounted 251 on a Leica Microscope (DM IL LED) at 20x magnification [30]. Assays were performed with 8 replicates per dilution.

### 2.5 Continuous culture assays

CB-CD34^+^ cells were plated with or without 20 μM ZOL at 10000 cells/well, in 48 well plates and then, at later time points of the experiment, re-plated in increasing amounts (plating at day 6 was 30000, day 12, 60000, day 21, 60000, and at day 27 100000 cells). The cells counts were taken using trypan blue B dye exclusion and cellular dilution factors were recorded and used to calculate the cumulative total cell numbers.

### 2.6 IC50 determination

The half maximal inhibitory concentration (IC50) values for ZOL inhibition in the different assays were calculated plotting experimental data in a dose response curve for non-linear regression analysis using GraphPad Prism V5.03 software.

### 2.7 Flow cytometry and apoptosis

CB-MA9 cells were stained with a PE-conjugated antibodies for CD11b (130-091-240), CD19 (130-091-247), CD33 (130-091-732), CD34 (130-081-002), CD38 (555460), CD44 (130-095-180), CD133/1 (130-080-801), or CD184 CXCR4 (130-098-354) (Becton Dickinson) by incubating with each antibody for 30 min, in the dark, at 4 °C and washed with PBS. Cells were re-suspended in 300 μl of PBS, acquired on the BD FACscan™ II and data were analyzed with FlowJo software 8.8.6 (Becton Dickinson) [36, 37]. Apoptosis was evaluated using the Kit from BioLegend (640930), APC Annexin V apoptosis detection Kit with 7-AAD, according to instructions.

### 2.8 RNA isolation, reverse transcription, and quantitative RT-PCR

TRI Reagent was used for RNA extraction (Sigma-Aldrich). cDNA was synthesized from 1 μg RNA using SuperScript III reverse transcriptase at 42 °C and 2.5 μM random hexamers (Life Technologies). Quantitative RT-PCR (Q-RT-PCR) was performed using the SYBR™ Green master mix (Bio-Rad) and the iQ5 multicolor detection system (Bio-Rad). One cycle of 3 min at 95 °C was followed by 45 cycles of 10 s at 95 °C, 10 s at 60 °C and 20 s at 72 °C, followed by a melting curve. mRNA levels are normalized to GAPDH mRNA expression and 2^−ΔΔCt^ values calculated [38, 39].The primer sequences used are: MA9 break-point, fwd: CACCTACTACAGGACCGCCAA rev: CTAGGTATGCCTTGTCACATT, HOXA9, fwd: CCCCATCGATCCCAATAACC rev: TCACTCGTCTTTTGCTCGGTC, MEIS1, fwd: AAGCAGTTGGCACAAGACACG rev: TGTCCATCAGGATTATAAGGTGTTCC and ZNF521, fwd: TGGGATATTCAGGTTCATGTTG rev: ACTGGAGTTTGGCAGGAGAG.

### 2.9 Total protein extraction

CB-MA9 cells cultured in suspension in the presence of ZOL (20 μM), geranylgeranyl pyrophosphate (GGPP 10 μM, Sigma), farnesyl pyrophosphate (FPP 10 μM, Sigma) alone or in combination. After 24h cells were collected and the whole-cell lysates were prepared. Cells were resuspended in lysis buffer 250 mM Tris-HCl pH 7.5, with protease inhibitors (Sigma) and subjected to three freezing and thawing cycles (80 °C–37 °C). The lysates were centrifuged at 12000 rpm for 10 min at 4 °C and the supernatant was recovered and quantified. Protein concentrations were determined by the Bradford assay at 595 nm using a standard curve prepared with bovine serum albumin (BSA, Sigma).

### 2.10 Western blotting

Whole cell extracts were fractionated on 4–12% NuPAGE Novex bis-Tris gradient polyacrylaminde gels (Life Technologies) under reducing conditions and electro-blotted onto nitrocellulose. After incubation with 5% Blotto in PBS-0.05%Tween20 for 30 min, the membranes were washed with PBS-Tween and incubated with antibodies in 5% Blotto in PBS-0.05%Tween20 at 4 °C for 24h. Antibodies used were Rap1A unprenylated (1:1000) (C-17 goat antibody, SC-1482 Santa Cruz Biotechnology), Rap1A/Rap1B (26B4) rabbit antibody (#2399, Cell Signaling Technology) (1:2000) and anti actin, mouse antibody (1: 2000, Sigma A4700). Membranes were washed with PBS-Tween and incubated with a 1:2000 dilution of HRP-conjugated secondary antibodies for 2h. Blots were washed with PBS-Tween and developed with ImmunoCruz Western blotting luminal reagent (Santa Cruz Biotechnology) and by autoradiography [40].

### 2.11 Statistics

All results are reported as mean ± standard deviation (SD). Statistical analysis was performed using t-test (∗p < 0.05 were considered statistically significant).

## 3 Results

### 3.1 Evaluation of cytotoxic effects of ZOL on CB-MA9 leukemic cells

The leukemic gene rearrangement MA9 was introduced into CB-CD34^+^ cells by lentiviral transduction and the cells were cultured in myeloid proliferation conditions with the cytokine cocktail (SCF, FLT3-L and IL3). These CB-MA9 cells become practically immortalized outgrowing the other normal hematopoietic cells [28] and a homogenous population of CD33/GFP positive cells was obtained by 40 days of expansion. Assays with ZOL were performed with cells after 50–60 days of proliferation.

**Figure 1:**
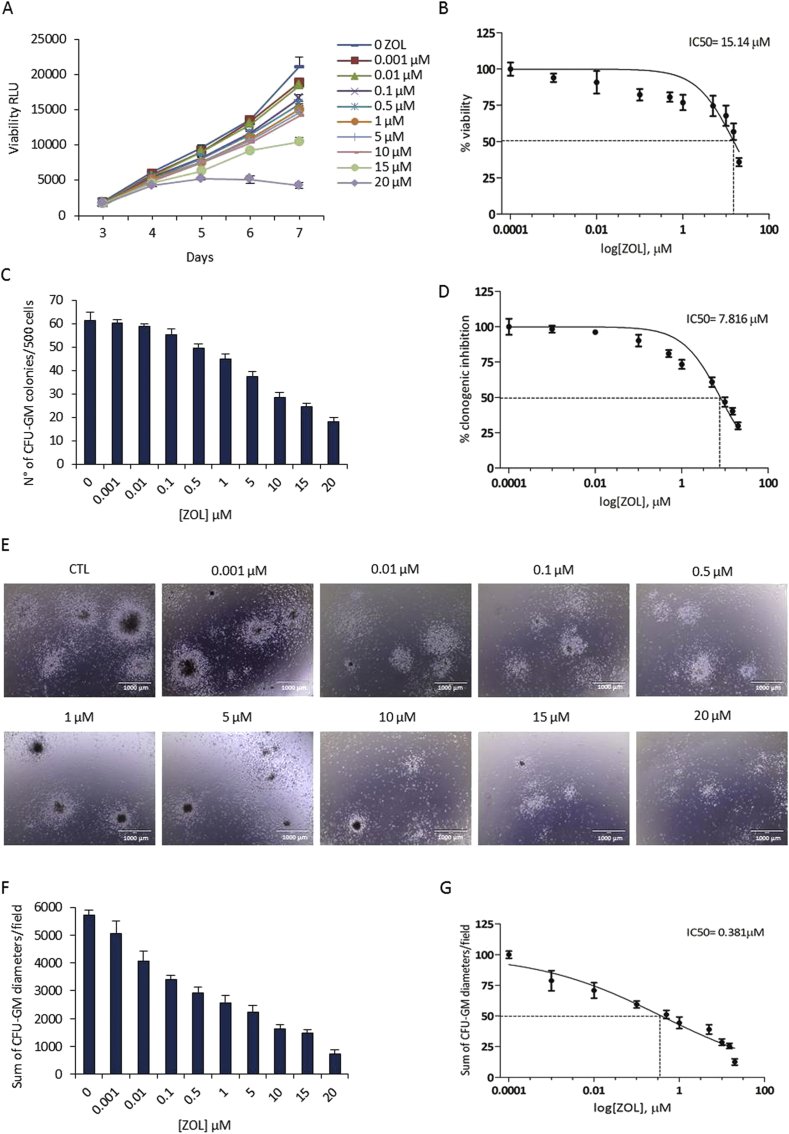
ZOL inhibits the proliferation and colony formation of CB-MA9 transduced cells. (A) CB-MA9 cells were seeded at 1 × 10^3^ cells/well in 96 well plates in quadruplicates and treated with increasing concentration of ZOL (0.001–20 μM). The viability was determined by the RealTime-Glo MT Cell Viability assay and represented as mean values ±SD. (B) The half-maximal inhibitory concentration (IC50) was calculated. (C) CB-MA9 cells were plated in methylcellulose with increasing concentrations of ZOL (0,001–20 μM) and colonies were counted after 14 days. (D) Dose-response curve for colony number IC50 value. (E) Images from a representative experiment (10x magnification, scale bar 1000 μm). (F) Colony sizes were quantified as diameter measurements and the sum of which per field is indicated. (G) Dose-response curve for colony diameter IC50 value.

Cell proliferation in the presence of ZOL was assayed in CB-MA9 transformed cells. In Figure 1A, it is shown that in suspension growth the cell viability, quantified by the RealTime-Glo MT cell viability assay remained high (>85%) at low concentrations of ZOL (0–1 μM), whereas at higher concentrations of ZOL (5–20 μM) cell viability was notably affected, with an IC50 value of 15.14 μM (Figure 1B).

The effect of ZOL on colony formation was evaluated on these CB-MA9 transformed cells *in vitro*. After 14 days of 3D culture in methylcellulose, there was a reduction in the number of Granulocyte-Macrophage Colony Forming Units (CFU-GM) in a dose dependent manner (Figure 1C) with an IC50 of 7.81 μM (Figure 1D). When considering the colony sizes (examples of which are shown in Figure 1E) and analyzed for diameter measurements (Figure 1F) the sensitivity to ZOL was notably increased with an IC50 of 0.38 μM (Figure 1G). This may reflect a higher sensitivity when cell-cell interactions in three dimension are required.

### 3.2 ZOL inhibits cobblestone area formation in an *in vitro* co-culture system of transformed HSCs and MS-5 cells

**Figure 2:**
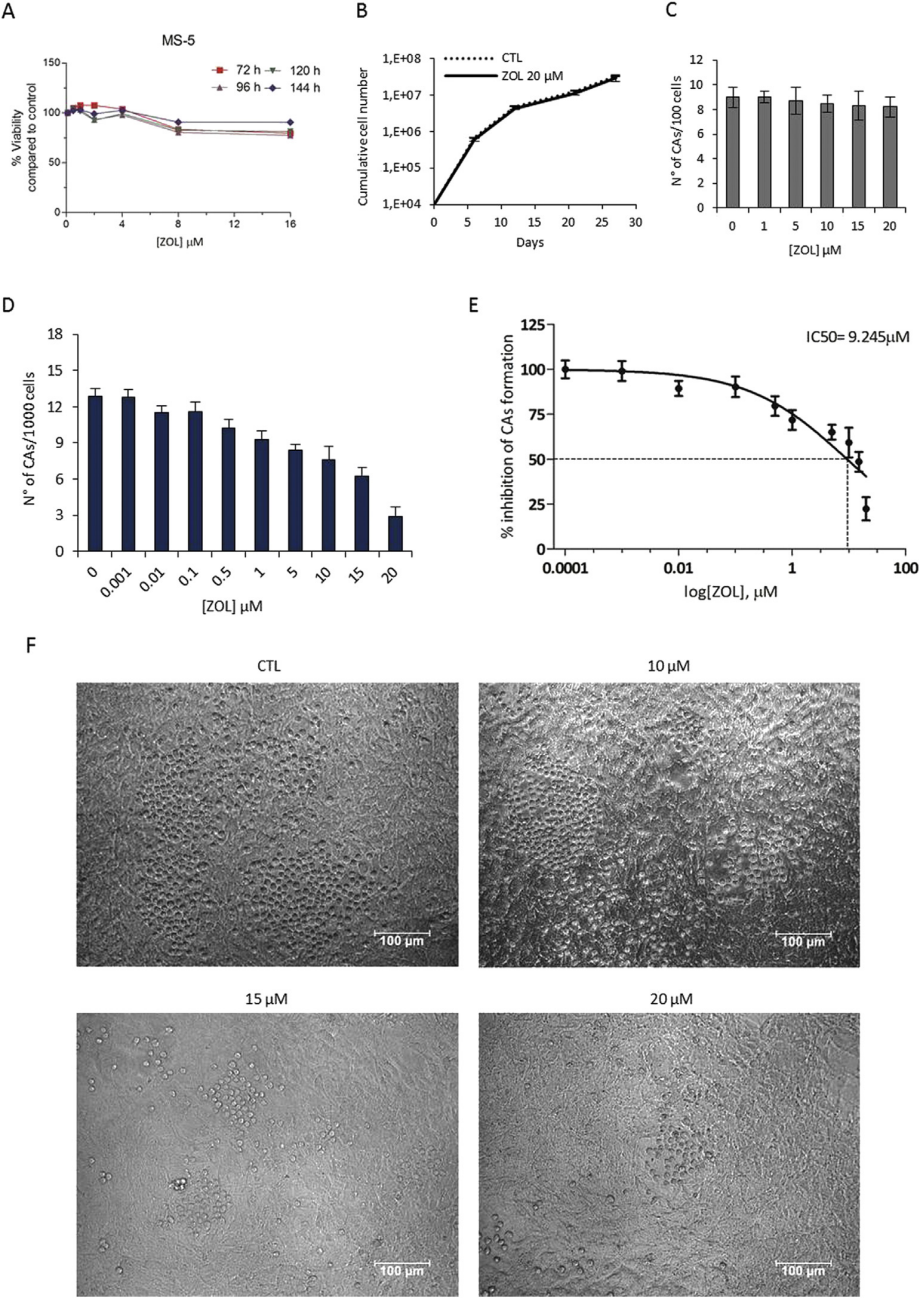
MS-5 stromal cells and CB-CD34^+^ hematopoietic stem cells are resistant to ZOL treatment whereas CB-MA9 cells in co-cultures are sensitive. (A) MS-5 stromal cells were treated with ZOL and the viability was measured by the RealTime-Glo MT Cell Viability assay. The percentage of viability was calculated normalizing values to control samples. (B) CB-CD34^+^ hematopoietic cells were treated either with or without 20 μM of ZOL for 27 days and cumulative cell numbers calculated. (C) CB-CD34^+^ cells were co-cultured on MS-5 stromal layers and exposed to ZOL (0–20 μM). After 6 days early CAs were optically counted. (D) CB-MA9 cells co-cultured on MS-5 cells in the presence of increasing ZOL (0.001–20 μM) and CAs counted after 6 days. (E) Dose-response curve for co-cultures of MS-5 cells and CB-MA9 cells for IC50 value of CAs number. (F) Representative images of co-cultures of CB-MA9 transduced cells with MS-5 in presence of 10, 15 and 20 μM ZOL (20x magnification, scale bar 100 μm).

In order to evaluate whether ZOL specifically targets the CB-MA9 cells, we assessed the effect of this drug on the MS-5, a stromal cell line which can support the growth of HSCs and on CB-HSCs purified CD34^+^ cells. Normal HSCs as well as leukemic transformed cells can be grown as co-cultures with the MS-5 stromal cell line where they form cobblestone like structures of cells which have burrowed underneath the stromal cells. When the MS-5 stromal cells alone were exposed to ZOL for up to 6 days relatively little growth inhibition was observed Figure 2A. CB-CD34^+^ hematopoietic stem cells were cultured for cumulative cell growth in presence or absence of ZOL (20 μM); throughout the proliferation culture there was no significant difference in cell viability compared to untreated cells (Figure 2B). Additionally when CB-CD34^+^ cells were plated on a MS-5 stromal layer the number of cobblestones areas formed (CAs) was not substantially affected by ZOL even up to 20 μM (Figure 2C). These results indicate that ZOL has little impact on a normal *in vitro* hematopoietic microenvironment.

The cobblestone area forming cell assay was performed to test the effect of ZOL on CB-MA9 transformed cells with the supportive MS-5 stromal cells. The assay with ZOL was evaluated by counting the number of CAs (Figure 2D) and gave a notable inhibition with an IC50 value of 9.24 μM (Figure 2E) and typical images where the size of the structures diminishes with ZOL are shown in Figure 2F.

### 3.3 Effect of ZOL on CB-MA9 cells

**Figure 3:**
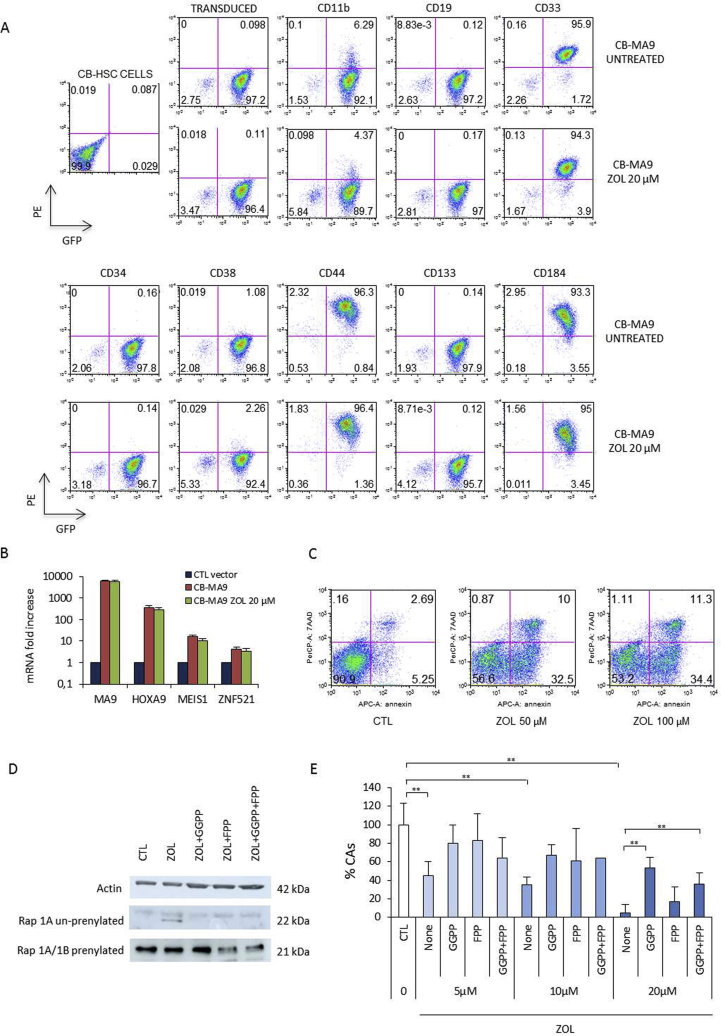
Effect of ZOL on CB-MA9 cells. (A) Cell surface antigens were evaluated with or without ZOL (20 μM) after 48 h by FACS analysis, (B) mRNA for the MLL-AF9 fusion protein and its targets HOXA9, MEIS1 and ZNF521 were quantified by RT-PCR after 48 h of ZOL (20 μM) treatment. (C) After 48 h with or without 20 μM ZOL treatment CB-MA9 were tested for early and late apoptosis. (D) CB-MA9 cells were cultured for 24 h in the presence or absence of ZOL 20 μM, with the addition of isoprenoid precursors GGPP 10 μM and FPP 10 μM (alone or in combination). The proteins were analyzed by Western blotting using antibody specific for unprenylated Rap1A compared to an antibody for both prenylated Rap1A/1B. Anti Actin was used as a control (Supplementary fig 1). (E) CB-MA9 cells were co-cultured with the MS-5 stromal monolayer and treated with ZOL (5, 10 and 20 μM) and GGPP 10 μM and/or FPP 10 μM. The reverted inhibition was quantified by enumerating the early CAs at 6 days.

CB-MA9 cells (derived from 60 days of culture) were treated with 20 μM ZOL for 48 h and then tested for cell surface markers. The CB-MA9 cells are over 95% positive for GFP, being a homogenous transduced population of continuously growing cells which has out grown other hematopoietic cells from the original transduction. These cells are predominantly of myeloid precursor phenotype (CD33^+^) rather than B cells (CD19^-^) with only a small component of CD11b. They no-longer express stem cell markers CD34, CD38, or CD133. A notable expression of the adhesion antigen CD44 as well as the CXCR-4 receptor for the chemokine SDF1 (CD184) are found. These cell surface antigens were not significantly modified after ZOL treatment (Figure 3A).

Analysis of mRNA levels (Figure 3B) confirmed the lentiviral introduction of the MLL-AF9 fusion gene by amplifying across the break point. The CB-MA9 cells (immortalized population 60–65 days) were compared to control transduced cells (control vector UMG-LV6) at 3 weeks when proliferation was still evident after this time point these cells no longer thrive. There was an evident increase in mRNA for the MA9 targets HOXA9, MEIS1 and ZNF521 [26-28] which was not affected by ZOL treatment.

ZOL is known to result in apoptosis and in these CB-MA9 cells early apoptosis, Annexin V as well as late apoptosis (7-AAD) were detected after 48 h (50 μM ZOL) (Figure 3C).

ZOL inhibits prenylation of GTPases including Rap1 in BCR-ABL leukemia [16]. Using an antibody specific for the unprenylated form of Rap1 it was found in CB-MA9 cells treated with 20 μM ZOL prenylation was inhibited and the unprenylated form could be detected. This inhibition could be reversed by exposition to the isoprenoid precursor molecules derived from GGPP (10 μM) or FPP (10 μM) or a combination of GGPP and FPP (Figure 3D) such that the unprenylated form of Rap1 was no longer detected.

The effect of the two isoprenoid metabolites was also validated in cobblestones area formation assays (CAs). As expected, CAs formation was reduced in CB-MA9 cells in a dose dependent manner with ZOL. Both FPP and GGPP alone or in combination were able to revert this inhibition. Cells were treated with different concentrations of ZOL and this reversal was especially evident at higher concentrations of ZOL (Figure 3E). This confirms the action of ZOL in inhibiting farnesyl diphosphate synthase (FDPS) required for prenylation in CB-MA9 leukemic cells.

## 4 Discussion

Zoledronic acid is currently used in the clinic to treat osteoporosis and bone metastases [1, 2]. Experimental studies have shown that ZOL also has an anti-tumor activity against a variety of cancer cells including leukemic cells assayed *in vitro* [11, 16, 17, 18]. The pharmacokinetic profile of ZOL however is such that it is rapidly eliminated from plasma due to renal excretion and retention in bone tissues [41] making it difficult to achieve a concentration capable of exerting a general anti-leukemic effect. If high concentrations of ZOL are used in the clinic there is the risk of osteonecrosis [41, 42]. However if specific types of tumor cells are identified that are uniquely sensitive to ZOL they may represent therapeutic targets. Here we have determined that the leukemic model transformed by the oncogenic fusion protein comprising MLL and AF9 is especially sensitive to ZOL, in proliferation viability assays as well as in clonogenic and stromal co-culture growth. A variety of different hematopoietic/leukemic cell lines have been tested for inhibition of cell growth by ZOL (Table 1) [[11], [12], [13], [14],^16^,^18^,^43^], allowing for the different methodologies used to assess the effect on viability ranges from 3-1100 μM to achieve 50% of inhibition IC50.

**Table 1:** Inhibitory effect of Zol in hematopoietic cell lines.

Cell line	Leukemic lineage	Viability Assay	IC50 ZOL (μM)	Reference
BV173	Pre-B cell erythroleukemia (Ph^+^)	Trypan blue dye exclus. (48h)	24.1	[16]
K562	Erythroleukemia		60.9	
HL60	Acute myelogenous leukemia		87.2	
NALM6	Pre-B cell ALL		73.4	
SEM	Biphenotypic acute leukemia		26.1	
AR230	Chronic myeloid leukemia	MTS (72h)	52.9	[18]
KCL22	Chronic myeloid leukemia		102.7	
HL60	Acute promyelocytic leukemia	WST-1	1050 (24h)	[11]
			398 (96h)	
K562	Erythroleukemia	XTT	60 (48h)	[43]
			42 (72h)	
NB4	Acute promyelocytic leukemia	MTS	100 (72h)	[13]
HL60	Acute promyelocytic leukemia	WST-8	1100 (48h)	[12]
			410 (72h)	
CIR	B cell lymphoma	CellTiter- Glo (96h)	19	[14]
RAMOS-RA1	Burkitt's lymphoma		100	
Daudi	Burkitt's lymphoma		240	
Raji	Burkitt's lymphoma		63	
RPMI8226	Multiple myeloma		3.5	
HL60	Acute promyelocytic leukemia		460	
NOMO-1	Acute myeloid leukemia		28	
SCC-3	monocyte-like leukemia		13	
THP-1	Acute monocytic leukemia		14	
U937	Histiocytic lymph		16	
P31/FUJ	Acute myeloid leukemia		65	
K562	Erythroleukemia		2.9	
J.RT3-T3,5	T cell acute lymphoblastic leuk.		20	
MOLT-3	T cell acute lymphoblastic leuk.		74	
MOLT-4	T cell acute lymphoblastic leuk.		71	
PEER	T cell acute lymphoblastic leuk.		55	
CB-MA9	Acute myeloid leukemia	RealTime Glo-luc	15.15	present manuscript
		Colony assay Nr	7.81	
		Colony assay diameters	0.38	
		Cobblestone areas Nr	9.24	

Testing CB-MA9 cells in suspension cell viability assays gave a relatively low IC50 of 15 μM. When the MA9 cells were cultivated as colonies in a 3D structure they displayed a greater sensitivity to ZOL (IC50 7.8 μM) for colony number and sensitivity was even more evident when considering colony size (IC50 0.38 μM). At 50 μM ZOL early and late apoptosis was evident. Additionally in co-culture assays with MS-5 cells CB-MA9 cells the cobblestone formation was notably sensitive to ZOL compared to normal CB-HSC CD34^+^ cells.

Cell-cell interactions are dependent on the complement of proteins on the cell surface the majority of which are conjugated with carbohydrates or lipids. ZOL is known to effectively [3] inhibit the FDPS enzyme required for farnesylation and prenylation particularly of GTPases. Without the hydrophobic prenylation modification the GTPases are unable to associate to the membrane and accumulate in the cytoplasm such that they escape Ras/Rac-GTPase signaling. In osteoclasts, they are known to function in the cytoskeletal organization, membrane ruffling, trafficking of intracellular vesicles, motility and apoptosis [3, 31].

Mouse models for the study of MLL-AF9 induced by either directly retro transduced human cord blood CD34^+^ cells or after cell line establishment have been used [44]. It was shown that using mice NOD/SCID as well as those transgenic for the human cytokines SCF,GM-CSF and IL3 that they were able to develop leukemias with a shortened latency compared mice without human cytokines. It had been noted that Rac1 and Cdc42 mediators of cell migration and engraftment were increased in murine MLL-AF9 LSC cells [45] by amount and activation with GTP. The Rac inhibitor NSC23766 [44] which blocks the guanine exchange factor (GEF), was found to inhibit proliferation, results in cell cycle arrest and apoptosis of MLL-AF9 cells with little effect on non-transduced CD34^+^ cord blood cells. The involvement of Rac signaling was confirmed by silencing of Rac1 affecting MA9 and not non transduced normal cord blood cells.

The use of MLL-AF9^+^ cell lines has permitted an analysis of the mechanism of action of Rac signaling. Experiments with the MLL-AF9 positive cell lines (THP-1, MM6) compared to MLL-AF9 negative cell lines (NOMO-1, HL60, Jurkat) have also been shown to be more sensitive to the mevalonate/cholesterol pathway by statin inhibition as well as the small molecule Rac inhibitors (NSC 23766 and EHT1864) [46]. The response to Rac inhibition resulted in caspase driven apoptosis, activation of caspase 8,9,7,3 and a notable reduction in pro-survival factors; survin, XIAP, and pAKT. This was preceded by phosphorylation of H2AX indicating DNA double strand breaks with genomic instability. Knock out mice for Rac1 or Rac2 [32] was sufficient to impair the survival and growth of MLL-AF9 leukemia and this could be rescued by Bcl-xl with the BH3-mimetric ABT-737.

In the present paper we show by using ZOL that inhibition of the mevalonate pathway at the stage of farnesylation (FDP synthase) compromises the growth of the CB-MA9 cells, particularly when the cells require membrane dependent cell-cell interactions in 3D colony culture. ZOL was also found to effectively inhibit the formation and size of cobblestone like structures which form when HSCs or MA9 cells migrate burrowing underneath the stromal layer. This aspect of migration is likely to be involve prenylated-GTP binding proteins which act to promote the formation of lamellipodia, sheet like membrane protrusions frequently found at the leading edge of migrating cells [47]. The inhibition of ZOL specifically for motility has been documented in the breast MDA-MB 231 cell line by cell tracking [48] and was found to require the focal adhesion kinase.

The CB-MA9 cells have a high degree of specific sensitivity to ZOL. In the co-culture growth of CB-MA9 they were found to be more sensitive to ZOL than M5-S stromal cells. Importantly normal HSCs displayed practically no response to ZOL treatment maintaining their expansion growth, colony formation and cobblestone capacity in the presence of a high (20 μM) concentrations of ZOL. This insensitivity for ZOL in CFU-GM colony formation assays was also found with normal HSCs obtained by leukapheresis [15, 16]. Also peripheral blood mononuclear cells from healthy donors have been found to be refractive to high ZOL concentrations even at 300 μM an IC50 value was not reached [49]. Indeed in normal conditions ZOL can instead increase the hematopoietic cell expansion through stimulation of the osteoblastic niche [19, 20].

The sensitivity to ZOL in leukemic cells compared to normal hematopoietic cells, may have developed with their oncogenic transformation with activation of tyrosine kinases, chemokine receptors, and intrigrins such that the various Ras/Rac/Rho GTPase signaling pathways are dysregulated determine proliferation and progression of the hematological malignancy. The loss of Rac1 in hematopoietic cells impairs engraftment, homing, localization and proliferation whereas loss of Rac2 results in reduced survival and retention in the bone marrow [50, 51]. The expression of constitutively active form of Rac1 (Rac1-V12) in HSPCs, promoted the retention of HSCs in the niche having higher CD44, VCAM-1, c-MPL, CXCR4, and N-cadherin, enhancing colony formation, quiescence and preventing leukemia cells from apoptosis [52] suggesting that inhibition of Rac GTPase activity could be an effective way of counteracting AML leukemias.

The GTPase family of proteins are functionally regulated by guanine nucleotide exchange factors (GEFs), GTPase activating proteins (GAPs) and guanine nucleotide dissociation inhibitors (GDIs). The GEFs replace the bound GDP with GTP activated signaling and GAPs enhance intrinsic GTPase activity. Control is due to extensive post transcriptional modifications; from prenylation for plasma membrane association, palmitoylation for targeting to detergent resistant membrane regions enhancing stability, as well as by phosphorylation, sumoylation and ubiquitination (reviewed by Durand-Onayl) [4]. The dependence of MA9 on activated Rac1/2 [44,45] led us to determine if it could be targeted by blocking prenylation through ZOL inhibition of farnesylation required for prenylation.

Zol has the advantage of being already approved for clinical use in the treatment of bone disorders. Considering the differential sensitivity of normal HSCs to MA9 cells it may be possible to target preferentially the leukemogenic cells before leaving the normal bone marrow niche for circulation. It is known that concentrations of ^14^C-ZOL once introduced into dogs rapidly accumulates in the bone, and was however- found to be 20 times lower in the bone marrow and practically absent in the peripheral blood [53]. Even so a relatively high concentration is likely to prevail in the hematopoietic niche which is considered to be in close proximity to the bone where normal HSCs and LSCs are thought to emerge, potentially giving a window for targeting before they are released from the niche.

To optimize the possibility to use ZOL as an anti-cancer reagent several strategies have been used involving a combination of drugs directed at different stages of the mevalonate pathway using Statins and ZOL in breast cancer cells [54, 55], ovary cancer [56] and in cerebral cavernous malformations [57] as well as using ZOL in combination with chemotherapy drugs [16, 17] in Ph^+^ primary leukemias and in cervical cancer [58]. The administration of ZOL to cancer patients prevents osteoporosis [1] as well as in metastatic bone disease acting on bone osteoclasts and resorption, the activity of ZOL being absorbed by the hydroxyapatite results in the control of hypercalcaemia in AML patients [5]. The consequence of blocking farnesylation by ZOL also results in the accumulation of the metabolic intermediate isopentenyl pyrophosphate which activates T cells through the Th1-like Vγ9Vδ2 T cell receptor for cytotoxic killing of cancer cells and leukemias [22, 59].

Promising protocols using nanoparticles and stealth liposomes for the delivery of ZOL can increase its effective concentration [60, 61] as well as the development of heterocyclic bisphosphonates which are considerably more active than ZOL [14] have been developed and need to be tested in *in vitro* and clinical models.

## 5 Conclusions

The anti-leukemic effects of ZOL has been evaluated in normal and leukemic cells using *in vitro* assays. CB-MA9 cells were especially sensitive to ZOL displaying inhibition in proliferation, clonogenic and stromal co-culture assays compared to normal HSCs and stromal MS-5 cells. The effect of ZOL inhibiting FDPS required for prenylation particularly of the small protein GTPases limits their motility and cell-cell interactions required for leukemic cell expansion and leukemogenesis. The MLL-AF9 transformed cord blood cells used in this model display a unique sensitivity for ZOL such that strategies combining chemotherapy for MLL-rearranged AML leukemias with ZOL could target the leukemic stem cell population emerging in the hematopoietic niche in proximity to bone osteoclasts where ZOL is sequestered.

## Declarations

### Author contribution statement

E. Chiarella, B. Codispoti and A. Aloisio: Performed the experiments; Contributed reagents, materials, analysis tools or data.

E. Cosentino, S. Scicchitano, Y. Montalcini and D. Lico: Analyzed and interpreted the data.

M. Mesuraca and H. Bond: Conceived and designed the experiments; Analyzed and interpreted the data; Contributed reagents, materials, analysis tools or data; Wrote the paper.

G. Morrone: Conceived and designed the experiments; Contributed reagents, materials, analysis tools or data; Wrote the paper.

### Funding statement

This work was supported by J18C17000620006 DEMOCEDE, Italy. S. Scicchitano was supported by fellowship funds (PON03PE_00009_2 ICaRe, Italy). A. Aloisio, Y. Montalcini, and E. Cosentino were supported by the PhD Programme in Molecular and Translational Oncology and Innovative Surgical Medical Technologies, Italy.

### Competing interest statement

The authors declare no conflict of interest.

### Additional information

No additional information is available for this paper.

## Figures and Tables

**supplementary fig1 -D-20-01218:**